# Prevalence and factors associated with use of herbal medicines during pregnancy among women attending postnatal clinics in Gulu district, Northern Uganda

**DOI:** 10.1186/s12884-016-1095-5

**Published:** 2016-10-06

**Authors:** Richard Nyeko, Nazarius Mbona Tumwesigye, Abdullah Ali Halage

**Affiliations:** 1 Department of Paediatrics and Child Health, St. Mary’s Hospital Lacor, P.O Box 180, Gulu District, Uganda; 2 Department of Epidemiology and Biostatistics, Makerere University School of Public Health, Makerere University College of Health Sciences, P.O. Box 7072, Kampala, Uganda; 3 Department of Disease Control and Environmental Health, Makerere University School of Public Health, Makerere University College of Health Sciences, P.O. Box 7072, Kampala, Uganda

**Keywords:** Herbal medicines, Pregnancy, Maternal health services, Maternal mortality

## Abstract

**Background:**

According to World Health Organization (WHO) estimates, 80 % of the population living in rural areas in developing countries depends on traditional medicine for their health needs, including use during pregnancy. Despite the fact that knowledge of potential side effects of many herbal medicines in pregnancy is limited and that some herbal products may be teratogenic, data on the extent of use of herbal medicines by women during pregnancy in the study setting is largely unknown. We determined the prevalence and factors associated with herbal medicine use during pregnancy among women attending postnatal clinics in Gulu district, Northern Uganda.

**Methods:**

This was a descriptive cross-sectional study which involved 383 women attending postnatal care across four sites in Gulu district using quantitative and qualitative methods of data collection. A structured questionnaire was used to collect quantitative data while qualitative data were obtained using focus group discussions and key informant interviews. The selection of the study participants was by systematic sampling and the main outcome variable was the proportion of mothers who used herbal medicine. Quantitative data was coded and entered into a computerized database using Epidata 3.1. Analysis was done using Statistical Package for Social Scientists version 13, while thematic analysis was used for qualitative data.

**Results:**

The prevalence of herbal medicines use during the current pregnancy was 20 % (78/383), and was commonly used in the second 23 % (18/78) and third 21 % (16/78) trimesters. The factors significantly associated with use of herbal medicines during pregnancy were perception (OR 2.18, CI 1.02-4.66), and having ever used herbal medicines during previous pregnancy (OR 2.51, CI 1.21-5.19) and for other reasons (OR 3.87, CI 1.46-10.25).

**Conclusions:**

The use of herbal medicines during pregnancy among women in Gulu district is common, which may be an indicator for poor access to conventional western healthcare. Perception that herbal medicines are effective and having ever used herbal medicines during previous pregnancy were associated with use of herbal medicines during current pregnancy. This therefore calls for community sensitization drives on the dangers of indiscriminate use of herbal medicine in pregnancy, as well as integration of trained traditional herbalists and all those community persons who influence the process in addressing the varied health needs of pregnant women.

## Background

The use of herbal medicines is believed to be increasing in many developing and industrialized countries, yet little is known about their use and safety especially during pregnancy [1]. Herbal medicines in this case are defined as plant-derived material and preparations perceived to have therapeutic benefits, containing raw or processed ingredients from one or more plants [2], and include herbs, herbal materials, herbal preparations, and finished herbal products that contain parts of plants or other plant materials as actual ingredients [3]. It is estimated that 80 % of the population living in rural areas in developing countries depends on traditional medicine for their health needs [4, 5], including use during pregnancy. While use of herbal medicines in pregnancy vary considerably between countries, many of the same herbs are used [6]. Evidence from the African continent suggests wide variability in use of herbal medicines during pregnancy, from a high of about 68 % as reported in one Nigerian study [7] to a low of 12 % in another study [8]. In Lusaka, Zambia, 21 % of pregnant women seeking care in public health system used traditional medicines during pregnancy [9].

In Uganda, it is estimated that over 60 % of the population seek medical attention from traditional healers, a pattern which cuts across all social classes and education levels [4], where traditional herbal medicine is widely used for prevention, diagnosis and treatment of social, mental and physical illnesses [10]. Majority of patients in Gulu district, including pregnant women are suspected to use traditional herbal medicines for a number of ailments, but the actual burden of use of herbal medicines by women during pregnancy is still unknown. In the same breath, use of herbal medicine is believed to be a contributing factor to poor access to, and use of, maternal healthcare services, including antenatal care (ANC) services and health facility delivery that are aimed at reduction of maternal death, currently at 438 per 100,000 live births [11], indicating that Uganda is unlikely to meet the millennium development goal (MDG) 5. For instance, whereas over 90 % of pregnant women attend at least one ANC visit, only about 47 % attend the World Health Organization (WHO)-recommended four or more ANC visits during their pregnancy [12], and only 57 % give birth in a health facility [11], suggesting possibility of resorting to alternative methods of health care, including use of herbal medicines. According to a report by Lamorde et al. (2008) in a study of medicinal plants used for HIV/AIDS related conditions in Gulu and other selected Ugandan disctrics, the most frequently used herbal medicine plant species include *Aloe sp, Erythrina abyssinica DC, Sarcocephalus latifolius, Psorospermum febrifugum spach, Mangifera indica L, and Warburgia salutaris* [13].

Like modern pharmaceutical drugs, herbal medicines, however, have the potential to cause adverse effects [14]. The causes of such adverse reactions are diverse, including the use of inherently toxic herbal medicines or an overdose of herbs, conventional drug-herbal medicine interactions, and idiosyncratic reactions such as allergies [15]. Therefore, relying on herbal medicines during pregnancy instead of scientifically proven treatment can have serious consequences, suggested to include fetal distress and premature deliveries [16], intrauterine growth restriction and decreased fetal survival [17], and congenital malformations [18], among others. Also among the variety of biological properties of herbal medicines is the ability to contract the uterus thereby posing the risk of abortion [19]. Furthermore, concomitant use of herbal products with the pharmaceutical medications leads to drug interactions with resultant undesirable effects of increased toxicity and decreased efficacy.

Despite the fact that knowledge of potential side effects of many herbal medicines in pregnancy is limited [1, 20–22], and that some herbal products may be teratogenic [23–25], exposure to herbal products is frequent in pregnancy [26], often on a self-treatment basis [27]. Similarly, while pregnant women recognize the potential risks of drug usage during pregnancy, they do not realize that herbal products could also be toxic, premised on the implicit belief that herbal products, being natural, are necessarily safe [28]. The use of herbal medicines in general, and in pregnancy in particular has been contributed to by a number of factors, including the belief that herbal products are safe and general ease of access [29], lack of access to public health system [5] and high cost of modern healthcare, perceived low costs of herbal products, socio-demographic characteristics, and social and cultural influences, among others [30, 31]. On the other hand, women who seek abortion may deliberately use the frequently unsafe traditional herbal products to induce fetal loss [2]. For instance, in one study of unsafe abortion in rural Tanzania, almost half of the women who experienced an unsafe abortion had resorted to traditional providers and plant species were in these cases often used as abortion remedies [32]. This study therefore seeks to determine the prevalence and factors associated with use of herbal medicines during pregnancy among women attending postnatal clinics in Gulu district, northern Uganda.

## Methods

### Study setting

The study was conducted in four (4) selected health facilities in Gulu district, northern Uganda, with a population of 479,496 inhabitants [33]. The main economic activity in the district is subsistence agriculture, in which over 90 % of the population is engaged. The district has one government regional referral hospital, two private hospitals, two health centre IVs and thirteen health centre IIIs which provide maternal health services including postnatal care services. However, access to health services still remains a challenge in the district as a whole. Over 37 % of the population moves a distance of more than 5 km to reach health services. High levels of poverty and illiteracy, especially among women, is exacerbated by high prevalence of preventable diseases.

### Study design and sample size

This was a descriptive cross-sectional study, using both quantitative and qualitative methods, and involving 383 postnatal mothers attending postnatal clinics (PNC) within the study period. The sample size was calculated using the Kish Leslie formula [34], using the formula, *n* = z^2^pq/d^2^, where *n* = required sample size, z = standard normal value corresponding to 95 % confidence interval (1.96), *p* = estimated proportion of herbal medicine use among pregnant women, which in this case is 21 % [9], q = p-1, and d = absolute errors between estimated and true value (5 %). This was multiplied by a design effect of 1.5, giving a total sample size of 383. The study population consisted of women attending postnatal clinic in the facilities in Gulu district during the study period. Women who were critically sick at the time of the visit (5 respondents) and those who were not able to understand the questions because of language barrier (2 respondents) were excluded.

### Sampling technique

Multistage sampling technique was used to select first, the health facilities for the study, and later, the respondents from each facility selected. The health facilities were first grouped into three strata comprising hospitals, health center IVs and health center IIIs. The only public hospital together with the two private hospitals were all grouped as one stratum of ‘hospitals’. Simple random sampling was then used to select the desired number of facility from each stratum where two hospitals and one health center each from the health center IV and III strata were selected as sites for the study. The determination of the number of health facilities chosen from each stratum was purposive in order to keep the research within the scope. The selection of the study participants from each of the sampled health facility was done by systematic sampling until the required sample size was realized. According to a preliminary survey of records from the health facility postnatal registers, it was estimated that about 20 mothers attend the PNC daily in hospitals and health center IVs (HCIVs), while about 10 attend in health center IIIs (HCIIIs). We therefore recruited 6 participants each day from the hospitals and HCIVs, and 3 from health center III. Therefore, every third mother (20/6 and 10/3 for hospitals/HCIV and HCIII respectively) was selected for the study, with the first participant being picked at random from assigned numbers. The selected mothers were then introduced to the study in more details, including the working definition of herbal medicine, and informed consent obtained for participation in the study before enrollement.

### Study procedure and data collection

A pre-coded and pre-tested structured questionnaire to capture respondents’ demographic characteristics, obstetrics characteristics, herbal medicine use during pregnancy and associated factors, and characteristics of herbal medicine use was used to collect quantitative data, while qualitative data were collected from focus group discussions (FGD) and key informant interviews (KII) using FGD and KII guides respectively. The questionnaires and interview guides were written in English as well as translated and administered in the local language understood by the participants.

For qualitative data, three focus group discussions (FGDs) were conducted in the community involving women who had not been part of the quantitative study in order to get a local perspective of the subject matter. Eight mothers were included in each of the FGDs. The groups involved mothers in the age group 20–38 years excluding grandmothers and mothers-in-law who usually have great influence in this community, in order to allow free expression of views. The discussions were moderated by the researcher and recorded on tape as well as notes taken by a rapporteur. Four key informants comprising one village health team (VHT), one local council one and two midwives were selectively interviewed to get a broader perspective of the aspects of herbal medicine use during pregnancy.

### Statistical analysis

#### Quantitative data

Data were coded and entered into a computerized database using Epidata 3.1. Data were cleaned and analysis was done at three levels using Statistical Package for Social Scientists (SPSS) version 13 software package*.* In univariate analysis, categorical variables were summarized as proportions, while continuous variables as means, median and standard deviations (SD). Prevalence was calculated as the proportion of study participants who used herbal medicines, the denominator being all postnatal mothers enrolled in the study. In the bi-variate analysis, the chi-square test (for categorical variables) and student *t*-test (for continuous variables) were used to test if the factors among mothers who used herbal medicines during pregnancy were different from those among mothers who did not use herbal medicines. Odds ratios, with 95 % confidence interval (CI) was used to measure the strength of association between use of herbal medicines during pregnancy and individual, socio-cultural, obstetrics/maternal, and health systems factors.

Multivariable analysis using logistic regression, backward stepwise procedure was used to select variables to be included in the final model to determine the factors that were independently associated with use of herbal medicine during pregnancy. Included in the model at multivariable analysis were factors that were significant at bivariate analysis and those with scientific plausibility though were not significant. *P*-value <0.05 was considered for statistical significance. Results were summarized in bar graphs, tables, and texts.

#### Qualitative data

Qualitative information generated from the FGDs and Key informant interviews were analyzed manually using thematic analysis according to emerging themes. Transcribed data were coded and main emerging themes were identified and presented as text quotes.

## Results

### Socio-demographic characteristics

**Fig. 1:**
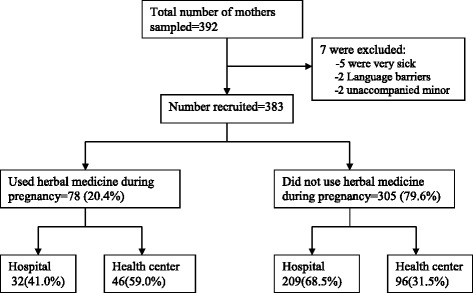
Study profile

Three hundred and eighty three respondents were enrolled in the study (Fig. 1). Majority, 41 % (156/383) of the respondents were between 20 and 24 years of age and at least 15 % (56/383) were teen mothers (15–19 year old), 70 % (39/56) of whom were in the legally recognized marriage age of 18–19 years. More than three-quarter of the respondents, 76 % (290/383) attained either no formal education or just primary level of education and majority 74 % (282/383) were residing in rural settings. The rest of the socio-demographic characteristics of the respondents are as summarized in Table 1. While all except two of the respondents attended antenatal care (ANC), only about half 49 % (186/383) had the recommended ≥4 goal-oriented ANC attendances. The other obstetrics characteristics of the respondents are summarized in Table 2.

**Table 1:** Baseline socio-demographic characteristics of the study population

Variable	Frequency (*N* = 383)	Percent
Mean age	25.32 (6.16)^a^	
Age group (years):		
< 34	339	88.5
≥ 35	44	11.5
Occupation:		
Peasant	292	76.2
Others	91	23.8
Marital status:		
Married	246	64.2
Not married	137	35.8
Education level:		
Tertiary	22	5.7
Below tertiary	361	94.3
Religion:		
Catholic	268	70.0
Others	115	30.0
Average monthly income (UGX):		
< 75,000	251	71.7
≥ 75,000	99	28.3
Residence		
Rural (outside the municipality)	282	73.6
Urban (within the municipality)	101	26.4
Distance from health facility		
> 5 km	169	44.1
≤ 5 km	214	55.9
Study site		
Gulu hospital	127	33.2
Lacor hospital	115	30.0
Awach HCIV	101	26.4
Bobi HCIII	40	10.4

^a^Mean (SD)

**Table 2:** Obstetrics and other characteristics of the study population

Characteristics	Number	Percent
Parity:		
Primiparous	87	22.7
Para ≥2	296	77.3
ANC attendance:		
Yes	381	99.5
No	2	0.5
^a^Number of ANC attendance:		
< 4 ANC	195	50.9
≥ 4 ANC	186	48.6
Place of delivery:		
Health facility	326	85.1
Home	57	14.9
Opinion about effectiveness & safety:		
Effective/safe	122	31.9
Not effective	261	68.1
^b^Used HM during previous pregnancies:		
Yes	121	35.7
No	218	64.3
Ever used HM for other reasons		
Yes	208	54.3
No	175	45.7

^a^Only the 381 respondents who attendend ANC, ^b^Only Para 2 and higher were considered

### Qualitative findings

The single most common theme that emerged from the qualitative data was facilitators of herbal medicine use, in addition to disclosure of herbal medicine use which also emerged as an issue to the respondents. The facilitator theme describes the factors that encouraged the use of herbal medicines and included belief that herbal medicines are effective in treating many ailments, previous experience with herbal medicines, cost of modern healthcare, and dissatisfaction with the healthcare system, among others. The disclosure theme mainly describes the respondents’ reasons for not volunteering information about use of herbal medicine to their healthcare providers and included fear of the health workers reaction, and being denied care. The two themes are described verbatim in quotes in triangulation with the quantitative data in the sections below.

### Prevalence of herbal medicine use

**Fig. 2:**
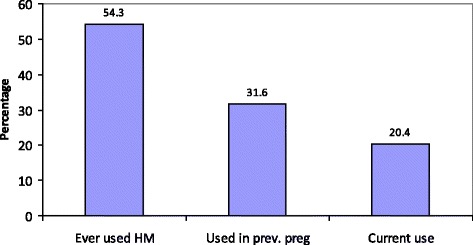
Prevalence of herbal medicine use by respondents

The prevalence of herbal medicine use during the current pregnancy was 21 % (78/383), while use of herbal medicines in general is common in the study setting (Fig. 2). Ingestion was the major method of use of herbal medicines 69 % (54/78) and majority 90 % (70/78) of the users of herbal medicines did not disclose the use of these local herbs to their attending healthcare workers at ANC and delivery (Table 3). Views during the FGDs did not differ much from the above finding, as reported by one of the mothers:

**Table 3:** Characteristics of herbal medicine use during pregnancy

Characteristics	Number (*n* = 78)	Percent
Trimester of pregnancy:		
1^st^ trimester	6	10.3
2^nd^ trimester	18	23.1
3^rd^ trimester	16	20.5
1^st^ and 2^nd^ trimester	10	12.8
2^nd^ and 3^rd^ trimester	11	14.1
During labour	9	11.5
Throughout pregnancy	6	7.7
Perception:		
Effective/safe	51	82.3
Not effective	11	17.7
Method of use:		
Ingestion	54	69.2
Smearing on abdomen	15	19.3
Insertion in vagina	6	7.7
Inhalation	3	3.8
Duration of HM use:		
< 3 week	57	73.1
≥ 3 weeks	21	26.9
Paid for herbal medicine:		
Yes	57	73.1
No	21	26.9
Source of herbal medicine:		
Gathered from bush	37	47.4
Traditional healer	39	50.0
Others	2	2.6
Source of information to use HM		
Relative	46	59.0
Herbalist	20	25.6
Friend	12	15.4
Would disclose use of HM to health worker		
Yes	20	25.6
No	58	74.4
Disclosed use of HM to health worker		
Yes	8	10.3
No	70	89.7
Concomitantly used HMs with ANC drugs		
Yes	50	64.1
No	28	35.9

*HM* herbal medicines, *ANC* antenatal clinic

*“For me I think it is not possible to tell the health workers that you took local herbs because they can become rude to you since they say people should come to hospital when they are sick but not to use local medicines. They can also refuse to give you treatment from the hospital if you say you have taken local medicines when you are also receiving care from the hospital.”* FGD, 20 year old mother.

Similarly, one of the key informants also observed that:

*“We sometimes get to know about these mothers using local herbal medicines during labour from the colour of their liquor or traces of the herbs in the genital parts, otherwise they will not tell you that they have used local herbs…”* KII, midwife.

Majority of users of herbal medicines during pregnancy had multiple reasons for use of these medicines, with the most common indications being abdominal/waist pain (43 %), febrile illness (45 %), inducing/enhancing labour (28 %), skin problems (24 %), nausea and vomiting (22 %), and difficult access to health facility (41.3 %), amomg others, as summarized in Table 4. The wide use of herbal medicines for various indications was also exemplified in one of the FGDs where one respondent asserted thus:

**Table 4:** Reasons for using herbal medicine during pregnancy

Characteristics	Number^a^	Percent^b^
Obstetrics reasons (*n* = 106):		
Nausea and vomiting	23	21.7
Abdominal/waist pain	45	42.5
Keep the baby healthy	8	7.5
Induce/enhance labour	30	28.3
Socio-cultural beliefs (*n* = 120):		
Tradition of use	43	35.8
HM cures many illnesses	47	39.2
Herbs are more efficacious	15	12.5
Herbs are safe in pregnancy	15	12.5
Health systems (*n* = 109):		
Low cost of herbal medicine	34	31.2
Difficulty accessing health facility	45	41.3
Lack of drugs in health facility	30	27.5
Concurrent illness (*n* = 105):		
Respiratory infections	29	27.6
Febrile illness	47	44.8
Skin problems	25	23.8
Other reasons	4	3.8

^a^Participants had multiple reasons for using herbal medicine

^b^percentage calculated using the denominator for each category

*“…local herbs work as well as other medicines. It works in waist pain, perianal wounds in children and “twor rubanga” (TB spine). I had a painful period and when I tried the hospital the medicines did not work but when somebody gave me local herbs, it subsided.”* FGD, 28 year old mother of four.

### Factors associated with herbal medicine use

At bivariate analysis, mothers who believed in herbal medicines were ten times more likely to use herbal products during pregnancy than those believing otherwise and this was statistically significant (OR 10.02; 95 % CI 5.67-17.73). This finding was supported by results from the FGDs where one of the respondents reported:

*“I was told that the local herbs are the ones made into modern medicines, so they should work the same way. I have used local herbs for all my ailments, including during my previous pregnancies and I have not experienced any problems. It is true people take local herbs for many illnesses and they get cured, sometimes even if you are taken to the hospital, the doctor can even say go back for local herbs because they cannot manage that condition.”* FGD, 35 year old mother.

Similarly, ever having used herbal medicines for other reasons was significantly associated with use of herbal medicines during the current pregnancy. The other individual and socio-cultural factors associated with use of herbal medicines among the respondents are as summarized in Table 5.

**Table 5:** Individual and socio-cultural factors associated with use of herbal medicines

Characteristics	Used HM *N* = 78 (%)	No HM use *N* = 305 (%)	OR (95 % CI)	*p*-value
Individual factors:				
Age in years:				
< 35	65 (19.2)	274 (80.8)	0.57 (0.28-1.14)	0.108
≥ 35	13 (29.5)	31 (70.5)	1.0	
Residence:				
Rural	64 (22.7)	218 (77.3)	1.82 (0.97-3.42)	0.059
Urban	14 (13.9)	87 (86.1)	1.0	
Religion:				
¶Catholic	58 (21.6)	210 (78.4)	1.31 (0.75-2.30)	0.344
Others	20 (17.4)	95 (82.6)	1.0	
Education level:				
Tertiary	4 (18.2)	18 (81.8)	0.86 (0.28-2.62)	0.793Ψ
Below tertiary	74 (20.5)	287 (79.5)	1.0	
Ever used HM:				
Yes	70 (33.7)	138 (66.3)	10.59 (4.93-22.77)	<0.001*
No	8 (4.6)	167 (95.4)	1.0	
Self-medication:				
Yes	67 (25.5)	196 (74.5)	3.39 (1.72-6.68)	<0.001*
No	11 (9.2)	109 (90.8)	1.0	
Income:				
< 75,000	57 (22.7)	194 (77.3)	1.32 (0.73-2.39)	0.353
≥ 75,000	18 (18.2)	81 (81.8)	1.0	
Socio-cultural factors:				
Occupation:				
Peasant	64 (21.9)	228 (78.1)	1.54 (0.82-2.91)	0.177
Others	14 (15.4)	77 (84.6)	1.0	
Marital status:				
Married	51 (20.7)	195 (79.3)	1.07 (0.63-1.80)	0.812
Not married	27 (19.7)	110 (80.3)	1.0	
Perception:				
Effective/safe	57 (46.7)	65 (53.3)	10.02 (5.67-17.73)	<0.001*
Not effective	21 (8.0)	240 (92.0)	1.0	

ΨFisher’s Exact Test, *P-value significant (<0.05), *OR* Odd’s ratio, *CI* = 95 % confidence interval, *HM* Herbal medicine. ¶ Catholic being the largest religious denomination in the district

Parity showed a statistically significant negative association with use of herbal medicines during pregnancy (OR 0.50; 95 % CI 0.25-0.98), implying women who are first time mothers were less likely to use herbal medicines during pregnancy. Women who used herbal medicines in their previous pregnancies were eight times more likely to use herbal medicines in their current pregnancies and this was statistically significant (OR 7.98; 95 % CI 4.45-14.30). Similarly, residing more than 5kms from the nearest health facility was more likely to be associated with use of herbal medicine during pregnancy and this was statistically significant (OR 2.43; 95 % CI 1.46-4.05) (Table 6). This finding was supported by results from the FGDs as typified by one respondent who, in expressing her concern, observed thus:

**Table 6:** Obstetric/maternal and health systems factors associated with use of herbal medicine during pregnancy

Characteristics	Used HM *N* = 78 (%)	No HM use *N* = 305 (%)	OR (95 % CI)	*p*-value
Obstetric/maternal factors:				
Parity:				
Primiparous	11 (12.6)	76 (87.4)	0.50 (0.25-0.98)	0.042*
Para ≥2	69 (22.6)	229 (77.4)	1.0	
Used HM in previous preg				
Yes	54 (44.6)	67 (55.4)	7.98 (4.45-14.30)	<0.001*
No	20 (9.2)	198 (90.8)	1.0	
ANC attendance:				
Yes	78 (20.5)	303 (79.5)	0.80 (0.76-0.84)	0.473Ψ
No	0 (0)	2 (100)	1.0	
Number of ANC:				
< 4	42 (21.5)	153 (78.5)	1.18 (0.72-1.96)	0.508
≥ 4	35 (18.8)	151 (81.2)	1.0	
Place of birth:				
H/Facility	61 (18.7)	265 (81.3)	0.54 (0.29-1.02)	0.055
Home	17 (29.8)	40 (70.2)	1.0	
Health system factors:				
Distance from HF:				
> 5 km	48 (28.4)	121 (71.6)	2.43 (1.46-4.05)	0.001*
≤ 5 km	30 (14.0)	184 (86.0)	1.0	
Costs of health services:				
Expensive	49 (23.2)	162 (76.8)	1.49 (0.89-2.49)	0.124
Not expensive	29 (16.9)	143 (83.1)	1.0	
Drug access in HFs:				
Available	42 (17.6)	197 (82.4)	0.64 (0.39-1.06)	0.080
Not available	36 (25.0)	108 (75.0)	1.0	

ΨFisher’s Exact Test, *P-value significant (<0.05), *OR* Odd’s ratio, *CI* 95 % confidence interval, *HF* Health facility, *HM* Herbal medicine

*“You can waste your little money and time going to the health facilities for problems like pain, cough, fever and the only thing they tell you is that there is no medicine, sometimes you only come back with aspirin or panadol, or you can even come back without any medicine and yet you travel from long distance which is expensive. So it is better to first get help from home and maybe only go to the health facility when you have failed to improve.”* FGD, a mother of five.

Simlilarly, one of the key informants from one of the health facilities reported:

*“…some of our mothers come from far distances to receive healthcare services and sometimes they are not able to afford transport money, so they prefer to use local herbs and only come to hospitals at the end…”* KII, midwife.

At multivariable logistic regression analysis, belief that herbal medicines are effective/safe, as well as previous use of herbal medicines during pregnancy or for other reasons were the only significant independent predictors of use of herbal medicines during pregnancy in this study. The factors that remained in the model even though were not statistically significant were age, distance from nearest health facility, self-medication and parity (Table 7).

**Table 7:** Multivariable logistic regression model for use of herbal medicines

Characteristics	AOR (95 % CI)	*p*-value
Age (<35/≥35 years)	1.05 (0.42-2.60)	0.915
Self-medication (Yes/No)	1.10 (0.46-2.61)	0.835
Perception (effective/not effective)	2.18 (1.02-4.66)	0.044*
Distance from health facility (>5 km/≤5 km)	1.45 (0.71-2.97)	0.310
Ever used herbal medicine (Yes/No)	3.87 (1.46-10.25)	0.006*
Used HM in previous pregnancy (Yes/No)	2.51 (1.21-5.19)	0.013*
Parity (para 1/para ≥2)	0.82 (0.32-2.05)	0.664

*P-value significant (<0.05), *OR* Odd’s ratio, *CI* 95 % confidence interval, *HM* herbal medicine

## Discussion

The present study determined the prevalence and factors associated with use of herbal medicines during pregnancy among 383 women attending postnatal clinics in Gulu district, Uganda. Our main findings show that use of herbal medicines during pregnancy in the study setting is common.

### Prevalence of herbal medicine use

The prevalence of herbal medicines use during pregnancy among the postpartum women in the current study is comparable to one reported by Yolan et al. (2007) in Lusaka, Zambia [9]. The current finding, however, contrasts with results from a similar study in Nigeria where a much higher proportion of women used herbal medicines during pregnancy [7]. The finding from the present study also shows a lower prevalence of use of herbal medicines during pregnancy in comparison with other similar studies [14, 35, 36]; but higher than the prevalence reported by Mothupi (2014) in Kenya [30] and by Gharoro and Igbafe (2000) in another Nigerian study [8]. This could be attributed to the differences in the populations studied, as well as differences in socio-cultural contexts, and health care systems (availability of services, access, and trust) in the different study settings. Furthermore, while Titilayo and colleagues (2009) looked broadly at use of herbal medicines in recent years among pregnant women [7], our study focused on current use, which renders plausible explanation to the likely higher percentage of use reported in their study as compared to that in the present study. Similarly, though Gharoro and Igbafe (2000) studied use of herbs among postnatal women [8], they only considered use of few specific herbs which is likely to explain the relatively lower prevalence of use. Similarly, the relatively lower prevalence of herbal medicine use during pregnancy found in the present study as compared to that from other studies could also be partly attributed to possible non-disclosure. This is supported by the fact that a significant number of respondents in the present study admitted that they would not disclose use of herbal medicine to the healthcare providers if they sought care from herbalists, a finding similar to that previously reported by Yolan et al. (2007) in Zambia [9].

### Factors associated with use of herbal medicines

Majority of the mothers who used herbal medicines during pregnancy perceived and believed that herbal medicines are effective and safe during pregnancy, a finding comparable to that by Azriani et al. (2008) in Malaysia [37]. This finding is also consistent wth previous findings by Mothupi (2014) in Nairobi, Kenya, were respondents used herbal medicine during pregnancy becasue of perception that western medicine was ‘not working’ and that herbal medicine was better or more effectice for their illness [30]. Similar findings have been reported by other authors [7, 31, 38], and could be explained by the fact that perception on the effectiveness of herbal medicines in solving problems will tend to influence whether mothers might use them again in the next pregnancy. More still, the above findings could also be related to cultural beliefs on causation of ill-health and belief that herbal medicines are inherently safe and cure many illnesses. Therefore, given that majority of the women do interface with the healthcare system at least once during their pregnancy, the healthcare providers should use this as an opportunity to sensitize them on the various limitations and potential adverse effects of herbal medicines, especially with regards to pregnancy.

Women who used herbal medicines during their previous pregnancies, as well as those who had ever used herbal medicines for other reasons were more likely to have used herbal medicines in their current pregnancies. This finding is consistent with that reported by Mothupi (2014) among postpartum women accessing public healthcare in Nairobi, Kenya [30], and suggests that the use of herbal medicines in previous pregnancy (cies) will likely predict their use in future pregnancies. The relatively common background use of herbal medicines in this setting as found in the present study was evidenced in one of the FGDs where six of the eight participants reported to have ever used herbal medicines for various other reasons. The above findings might be explained by the women’s previous experiences with, and belief that herbal medicines worked in solving their other problems, as well as to its longstanding integration into the culture and its perception as their own indigenous medicine.

There was a tendency to higher use of herbal medicines during pregnancy among women from rural areas (23 %) compared to their urban counterparts (14 %). Relatedly, women living farther away (more than 5kms) from any nearest health facility were twice more likely to use herbal medicines during pregnancy than those living less than 5kms from the nearest health facility, and this was statistically significant on bivariate analysis (p = 0.001), though did not independently predict use of herbal medicines. This could be attributed to challenges of accessibility of health services, and supports a previous report by Mothupi (2014) in Nairobi, Kenya [30], and Mbwanji (2012) in Tanzania [39] that long distance to the nearest health facility was a significant factor associated with use of herbal medicine during pregnancy. This finding may not be surprising since long distance from health facility hinders access to modern healthcare by increasing costs of transportation, as well as time to access health services. Herbal products, being readily available, cheap and provided by members who are well known in the community, therefore becomes an easier alternative. This implies that addressing challenges of availability and accessibility of healthcare services should be an important consideration in addressing the prevalent use of herbal medicines during pregnancy in this population.

### Indications and characteristics of herbal medicine use

Herbal medicines in the present study were used for multiple reasons including to relief symptoms such as fever, abdominal and waist pain, respiratory illnesses, and skin problems, as well as nausea and vomiting among others, a finding that resonates with that from several authors [7, 40, 41]. This finding has important implications given the fact that these are common symptoms which may indicate underlying serious complications during pregnancy such as severe malaria, or severe bacterial infections (urinary tract infections, pelvic inflammatory disease, septicaemia, or chorioamnionitis). This therefore calls for concerted health educations and community sensitizations as the use of herbal remedies may delay prompt diagnosis and effective treatment of these conditions with resultant adverse consequences to both the mother and fetus, including pregnancy wastage, a concern also previously highlighted by Kennedy et al. (2013) in a multinational study of herbal medicine use during pregnancy [6]. This scenario may also be important as a proxy indicator of lack of access to reliable healthcare services to the population, thereby calling for a thorough assessment of healthcare in the region.

Ingestion was the single most common method of using herbal medicine during pregnancy among the women in the study setting, with the other methods being smearing on the abdomen and insertion in the vagina, a finding similar to that by Kamatenesi and Oryem (2007) in a study in western Uganda [42]. These methods of usage, as found in the current and previous studies, may be attributed to the perceived actions and effects of the particular herbal products used, but with potential implications for interactions and interference with the routine orally ingested ANC medications. Similarly, the practice of particularly inserting herbal products in the vagina places these women at high risk of infections (genital tract infections), accidental rupture of membranes and pregnancy wastage. This finding therefore calls for concerted efforts in community sensitization in changing some of these potentially dangerious practices.

Herbal medicines in the study setting were mainly used during the second (23 %) and third (21 %) trimesters. Using herbal medicines during different trimesters of pregnancy may be associated with different effects, and shows great variation from one setting to another. For instance, in their study of herbal medicines use during pregnancy in western Uganda, Kamatenesi and Oryem (2007) found that herbal medicines were more commonly taken during labour (91 %) and in the third trimester (40 %), with very low use during the first trimester (7 %) [42]. In a Malaysian study, majority of the mothers took herbal medicines during the third trimester only (80 %), mainly to facilitate labour [37], a finding also reported by Rolanda and Sally (2006) in South Africa [43]. The variability in the timing of use of the herbal medicines could possibly be explained by the differences in the perceived desired effects and the reasons for use at a particular time; socio-cultural variability and belief, as well as differences in the types of herbs used by the different population groups. The use of herbal medicines during the first trimester as also found in the current study however raises concerns since this is the critical period of organogenesis, with potential risks for adverse effects like congenital anomalies.

A significant number of the users of herbal medicines during pregnancy in the current study used the herbal products concomitantly with the conventional medicines given during the antenatal care, a finding much higher than reported by Tabatabace (2011) in Iran [14]. In addition, more than half of the herbal medicine users during pregnancy reported to have not adhered to the conventional medicines dispensed during antenatal visits. This finding has an important policy implication as it presents a serious concern to components of the goal-oriented ANC interventions, especially prevention of maternal anaemia and malaria, as well as elimination of mother-to-child transmission when HIV positive expectant mothers do not adhere to antiretroviral drugs as a result of using herbal products. Furthermore, as suggested by Oshikoya et al. (2007) [44] and Chen et al. (2011) [45], concurrent use of herbal products with the conventional medicines as depicted in this study may result in interactions which may alter the pharmacokinetics of the drugs leading to an increase or decrease in plasma concentrations, thus altering the therapeutic outcomes.

### Limitations of the study

The study was conducted among postnatal mothers attending postnatal clinics which may have affected its generalizability since only a small proportion of mothers attend postnatal care. Similarly, the exclusion of emancipated minors who were not accompanied by adults is also likely to have affected the generalizability of the study findings. This study also included only two lower health facilities which may not be representative of the many health facilities at that level. However, attempt was made to overcome these shortcomings by triangulating the quantitative method with qualitative information collected within the local communities outside the health facilities. Information bias was also a likely problem since use of herbal medicines is very often perceived negatively by health workers, and hence some women may have feared admitting to use of herbal medicines.

## Conclusions

The use of herbal medicines during pregnancy among women in Gulu district is common, which may be an indicator for poor access to conventional western healthcare. The factors associated with use of herbal medicines during pregnancy include believe that herbal medicines are effective and safe, and having ever used herbal medicines during previous pregnancies and for other reasons. Many users have confidence in the efficacy of herbal medicines as an alternative treatment, with oral ingestion being the major method of use.

This therefore calls for community sensitization drives on the dangers of indiscriminate use of herbal medicine in pregnancy, as well as integration of trained traditional herbalists and all those community persons who influence the process in addressing the varied health needs of pregnant women.

## Abbreviations

ANC: Antenatal care
FGD: Focus group discussion
HC: Health center
KII: Key informant interview
SD: Standard deviation
UBOS: Uganda Bureau of Statistics
VHT: Village health team
WHO: World Health Organization
